# Predicting Hospital Overall Quality Star Ratings in the USA

**DOI:** 10.3390/healthcare9040486

**Published:** 2021-04-20

**Authors:** Nisha Kurian, Jyotsna Maid, Sharoni Mitra, Lance Rhyne, Michael Korvink, Laura H. Gunn

**Affiliations:** 1 Department of Public Health Sciences, University of North Carolina at Charlotte, Charlotte, NC 28223, USA; nkurian@uncc.edu (N.K.); jmaid@uncc.edu (J.M.); smitra3@uncc.edu (S.M.); lrhyne5@uncc.edu (L.R.); 2 School of Data Science, University of North Carolina at Charlotte, Charlotte, NC 28223, USA; 3 Westchester County Department of Health, White Plains, NY 10601, USA; 4 Welltok, Inc., Denver, CO 80202, USA; 5 ITS Data Science, Premier, Inc., Charlotte, NC 28277, USA; michael_korvink@premierinc.com; 6 School of Public Health, Faculty of Medicine, Imperial College London, London W6 8RP, UK

**Keywords:** hospital quality, star rating, performance measures, hospital compare

## Abstract

The U.S. Centers for Medicare and Medicaid Services (CMS) assigns quality star ratings to hospitals upon assessing their performance across 57 measures. Ratings can be used by healthcare consumers for hospital selection and hospitals for quality improvement. We provide a simpler, more intuitive modeling approach, aligned with recent criticism by stakeholders. An ordered logistic regression approach is proposed to assess associations between performance measures and ratings across eligible (*n* = 4519) U.S. hospitals. Covariate selection reduces the double counting of information from highly correlated measures. Multiple imputation allows for inference of star ratings when information on all measures is not available. Twenty performance measures were found to contain all the relevant information to formulate star rating predictions upon accounting for performance measure correlation. Hospitals can focus their efforts on a subset of model-identified measures, while healthcare consumers can predict quality star ratings for hospitals ineligible under CMS criteria.

## 1. Introduction

Choosing a hospital can be a difficult decision, especially when seeking a high-risk treatment or a life-saving procedure. In general, patients often make choices based on a hospital’s perceived reputation [1]. Patients in the United States (USA) can make decisions by using information from the Five-Star Quality Rating System for Hospitals [2]. This program, developed by the Centers for Medicare and Medicaid Services (CMS) and made publicly available in 2016, evaluates the overall performance of hospitals in the USA and assigns a rating to hospitals based on a one-to-five-star scale. Each hospital’s overall rating shows how well that hospital has performed as compared with other hospitals in the USA. This rating system was designed specifically to enable individuals to select and compare hospitals through a method that is easy to comprehend [2].

While the Five-Star Quality Rating System was created for healthcare consumers, it is also vital for hospitals that want to remain profitable, since high ratings attract more patients [3]. Thus, the five-star quality rating system encourages hospitals to maintain and improve the quality of services they offer to their patients. Most hospitals’ quality ratings are unimpressive, with the most common score being three stars as of January 2019 [2]. Hospitals can build on CMS’s quality star ratings to assess areas of potential improvement and implement changes to their practices, services, or facilities with an aim to improve their overall quality rating.

Quality is a multidimensional feature for hospitals [1]. CMS currently uses hospital-reported quality performance measures through the Hospital Inpatient Quality Reporting and Hospital Outpatient Quality Reporting programs to assess hospitals’ overall quality star ratings [2,4]. These performance measures can be obtained from Hospital Compare, a database that provides information on patient hospital care in the USA [5]. CMS collects information regarding 57 performance measures, which are categorized into the following seven domains: mortality, safety of care, readmission, patient experience, effectiveness of care, timeliness of care, and efficient use of medical imaging [2,4]. Performance measures are risk-adjusted, when necessary, to enable a fair comparison across facilities. These adjustments include pre-existing patient characteristics which could increase patients’ risks, such as past medical history, comorbidities, and patient condition at the time of arrival [6]. Then, a weighted summary score is used to determine the overall hospital quality star rating [6,7]. 

Hospital Compare compiles data regarding the quality of care at over 4500 Medicare-certified hospitals, excluding Veterans Health Administration and Department of Defense hospitals [2]. However, not all of these hospitals are eligible for a star rating. CMS defines star rating eligibility as those hospitals that have a minimum of three performance measures across at least three domains, including one measure domain of mortality, safety of care, or readmission [2]. While the premise of CMS’s star rating program is beneficial for patients, families, caregivers, physicians, and hospital administrators, a common criticism to the star rating methodology, through stakeholder input, is that it is overly complex [7] and suffers from instability when performance measure weights are shifted across measures simply based on latent correlations [8]. In 2019, CMS began considering an “explicit approach” in response to criticism [9], where a more interpretable and transparent model would be built, compared to the latent variable modeling approach that CMS currently uses [7]. Thus, part of the motivation of this study is to offer a more explicit, alternative methodological approach.

This study uses Hospital Compare data to determine a hospital’s predicted overall quality star rating, accounting for covariates across a range of inpatient and outpatient performance measures. A primary aim of this study is to identify performance measures with the strongest (negative or positive) impact on hospitals’ quality star ratings upon accounting for performance measure correlations. Hospitals can utilize this approach to focus their efforts on specific areas that may need attention and with potential cascade effects on other measures, in order to improve or maintain their overall quality star ratings. Furthermore, we offer a simpler, more explicit and intuitive methodological approach for predicting overall hospitals’ quality star ratings in the USA.

## 2. Materials and Methods

Hospital Compare data released in the spring of 2019 were retrieved for analysis. Data for the 57 performance measures were extracted from the March 2019 dataset release. Data collection periods varied for each measure domain [10]. Hospitals’ overall quality star ratings, the outcome of interest, was extracted from the April 2019 dataset release, since ratings were built on performance measures collected and released beforehand [10]. 

There were 4772 hospitals contained in the raw data. Six hospitals that were not included in the March file were removed as they did not have performance measures. An additional 247 hospitals were removed since they were not eligible to obtain an overall hospital quality star rating based on the aforementioned criteria. Thus, 4519 hospitals were eligible to receive a star rating. However, a further 805 star-rating-eligible hospitals were removed due to missing star ratings, resulting in 3714 hospitals among which analyses were performed.

Prior to removing hospitals with missing star ratings, Markov Chain Monte Carlo (MCMC) was used to impute missing covariate performance measure values across 4519 hospitals, regardless of star rating availability [11]. To validate the imputation technique, descriptive statistics were compared to the complete data prior to imputation. To address large variations in the scales of covariates, all performance measures were standardized. 

After removing eligible hospitals with missing star ratings, an ordinal logistic regression model with stepwise variable selection was implemented to identify performance measures associated with the overall hospital star rating. The reference category for the ordinal outcome was the star rating of five. Entry and removal significance levels for the stepwise variable selection were both set at alpha = 0.05. Ordinal logistic regression models were fitted with and without performance measures that were identified as highly correlated in order to assess the impact of multicollinearity on the model. Akaike information criterion (AIC) was used to determine the resulting model of analysis. Odds ratios provide a more intuitive assessment of the relationship between performance measures and star ratings. Finally, a comparison of the CMS approach and this final model was performed. Statistical Analysis System (SAS) version 9.4 was used for all analyses.

Inference on star ratings is possible for hospitals without a full set of reported measures, by using the fitted model to forecast the star rating after multiple imputation of performance measures that are not available. This is especially relevant for both star ineligible hospitals (who may be interested in understanding what their rating could be, if eligible) and those with missing star ratings, where healthcare consumers need absolute or relative quality assessments of those hospitals to compare their options for healthcare delivery.

## 3. Results

Table 1 provides descriptive statistics for hospitals’ overall quality star ratings among eligible hospitals (*n* = 4519) in 2019. Less than one-fifth (*n* = 805) of eligible hospitals were missing a star rating. Among hospitals reporting a star rating, the most common value was a star rating of three (*n* = 1258, 33.87%). Less than one-tenth of eligible hospitals reporting a rating received the lowest star rating of one (*n* = 281, 7.57%), and similarly for the highest star rating of five (*n* = 292, 7.86%). 

Missing values among performance measures across all 4519 star-rating-eligible hospitals ranged from 167 (3.7%) for hospital-wide unplanned readmission within 30 days (variable identifier READM_30_HOSP_WIDE) to 4055 (89.73%) for median time to transfer to another facility for acute coronary intervention (variable identifier OP_3b_2). 

Table 2 presents descriptive statistics, prior to standardization, for the 20 performance measures comprising the final model after variable selection. Some performance measures show large standard deviations, such as median time from emergency department (ED) arrival to ED departure (variable identifier ED_1b), which has a standard deviation of 109.75 min, or admit decision time to ED departure for admitted ED patients (variable identifier ED_2b), which has a standard deviation of 69.29 min, therefore, all measures were standardized, as mentioned in the Materials and Methods Section, for easier interpretability of the model results. 

Odds ratios and corresponding 95% confidence intervals (CIs) for the covariates in the resulting model are presented in Table 3, in addition to the CMS factor loading coefficients [12] for comparison. Table 3 also contains the full list of performance measures considered by CMS, which is also the complete list of measures included in our full model. One of these performance measures is the percentage of administration of aspirin on arrival to an emergency department (ED) for patients with acute myocardial infarction (AMI) or chest pain (variable identifier OP_4). As seen in Table 3, the corresponding estimated odds ratio is 1.14 (95% CI 1.05 to 1.23). Thus, for an increase in one standard deviation (6.60%) of administration of aspirin to AMI or chest pain patients on arrival to the ED, a statistically significant increase of 14% in the odds of observing a quality star rating of 5 (versus a rating of 1, 2, 3, or 4) is expected, while keeping all other covariates in the model constant. 

In another example, the 30-day mortality rate for patients with pneumonia (variable identifier MORT_30_PN) has an estimated odds ratio of 0.46 (95% CI 0.42 to 0.50). For an increase in one standard deviation (1.97) of the 30-day mortality rate for patients with pneumonia, a statistically significant decrease of 54% in the odds of observing a quality star rating of 5 (versus a rating of 1, 2, 3, or 4) is expected, while keeping all other covariates in the model constant.

## 4. Discussion

An ordinal logistic regression model is proposed to infer hospitals’ quality star ratings in the USA using a set of twenty relevant performance measures which have been identified through stepwise variable selection. Additionally, since these measures were assessed ahead of the ratings, causality is self-evident.

The example findings provided in the Results Section regarding administration of aspirin to AMI patients on arrival to the ED align with expectations, since early administration of aspirin is the recommended practice guideline for AMI patients [13].

The predicted effect of most of the performance measures in the ordinal logistic model aligns with the literature. For example, increases in the performance measures pertaining to 30-day mortality rates (variable identifiers: MORT_30_COPD, MORT_30_HF, MORT_30_AMI, MORT_30_STK, and MORT_30_PN) were predicted to significantly decrease the odds of observing a quality star rating of 5 (versus a rating of 1, 2, 3, or 4). Higher mortality rates have been associated with poor quality of care [14].

Larger values of measures associated with delayed care in an emergency department (variable identifiers: ED_1b, ED_2b, and OP_22) were also predicted to decrease the odds of observing an overall quality star rating of 5 (versus a rating of 1, 2, 3, or 4). Delayed care in the ED can lead to lower overall patient satisfaction and higher mortality rates [15].

Most performance measures pertaining to the Hospital Consumer Assessment of Healthcare Providers and Systems (HCAHPS) survey (variable identifiers: H_COMP_1, H_COMP_5, H_COMP_6, H_COMP_7, and H_HSP_RATING) were predicted to increase the odds of observing an overall quality star rating of 5 (versus a rating of 1, 2, 3, or 4). High HCAHPS survey scores have been associated with higher hospital ratings [16]. However, as per our model, upon accounting for all other covariates, an increase in HCAHPS Responsiveness of Hospital Staff (variable identifier: H_COMP_3) was associated with a decrease in the odds of observing a star rating of 5 (versus a rating of 1, 2, 3, or 4). This was the only statistically significant performance measure in our model with results that were unexpected, which could be due to that effect being captured by other covariates related to communication, such as nurse communication (variable identifier H_COMP_1).

When comparing the loading coefficients used in the CMS model, which uses a latent variable modeling approach not accounting for performance measure correlations, to the odds ratios in our model, which are presented in Table 3, some similarities are found with respect to relative importance of various performance measures on the overall quality star rating outcome. Within each performance measure domain, the variable with the largest CMS loadings was statistically significant in our ordinal logistic model. For example, the performance measure with the largest contribution within the mortality domain was 30-day mortality rate for heart failure (variable identifier MORT_30_HF). It can also be seen that most of the loading coefficients with a value approximately equal to or greater than 0.5 in the CMS model appeared in our ordinal logistic model, with the exception of performance measures in the patient experience and timeliness of care domains. This can be attributed to substantial multicollinearity present within these domains. There are strong correlations (Pearson correlation coefficient ≥0.7) among a number of covariates within the patient experience domain, as well as median time from ED arrival to ED departure for admitted ED patients (variable identifier ED_1b) and admit decision time to ED departure time for admitted patients (variable identifier ED_2b) within the timeliness of care domain. This results in ‘double counting’ of information in the CMS approach which is avoided in the model proposed in this manuscript, since CMS constructs their latent variable models in parallel for each measure domain, but ignores intergroup correlations among performance measures, which is a great source of statistical learning.

The approach described in this manuscript unveils a set of influential performance measures that contain the relevant information regarding hospitals’ overall quality star ratings in the USA. It reduces the double counting of information embedded when considering highly correlated performance measures across measure domains and provides a more intuitive link between performance measures and star ratings through odds ratios rather than latent constructs. Hospitals can focus their efforts on model-identified key measures and assess, through odds ratios, the expected changes in ratings upon improvements in those performance measures. Additionally, the proposed method allows for inference when all performance measures are not available, through multiple imputation. Imputation allows for overall quality star rating comparisons, and also allows for inter-hospital comparisons of performance measures that may not be readily available by healthcare consumers and providers, such as those relating to new hospitals or ineligible ones under CMS criteria. This is a first step toward a larger and needed healthcare discussion about providing a simpler, more intuitive approach than the use of latent variable modeling, through the use of odds ratios as an alternative, to assess relationships between hospitals’ performance measures and overall quality star ratings. 

This method does not rely on the nature or source of the covariates, but on how relevant they are to define the outcome metric of interest. CMS has recently modified the star rating system as part of a larger overhaul of metric refinements [17]. Beginning in 2021, these changes will include, for example, modifications to their latent variable approach and grouping of factors [18] and an attempt at enhancing interpretability of the information by healthcare consumers [19]. While we should expect additional modifications in the coming years to the star ratings system, those modifications may still rely on a structurally overly complex approach for which intuitive alternatives can benefit both sides of the supply and demand of healthcare. This may require future recalibrations of our proposed approach to align with the dynamism of those changes.

### Limitations

The proposed approach relies on a U.S.-centered metric. Other countries may rely on different metrics and factors to evaluate the quality of healthcare facilities. Therefore, this new approach cannot be easily extrapolated to other healthcare systems or countries. Some performance measures had large amounts of missing or non-reportable data, such as median time to transfer to another facility for acute coronary intervention (variable identifier OP_3b_2), which was missing for 89.71% of hospitals. Imputation was performed on all of the variables, including those that had large amounts of missing or non-reportable data. The values imputed for the performance measures are not observed clinical values. This could possibly lead to additional uncertainty in results [11]. However, these performance measures have lower factor loadings, and the high absolute level of correlation found across measures (intra- and inter-domain) further reduces the impact of imputation of missing data. Additionally, while this approach reduces the amount of information double counting in the original set of factors used by CMS, it does not completely remove it. Finally, this model does not intend to replace or offer an enhanced alternative to CMS’s star rating system. Our approach still relies on CMS’s outcomes (a by-product of their model and weights) to formulate a simpler, more intuitive version of the model that facilities and consumers can use.

## Figures and Tables

**Table 1:** Summary of hospitals’ overall quality star ratings.

Hospital Star Rating	*n*	Percent (%)	Cumulative Frequency	Cumulative Percent among Those Reporting Ratings (%)
1	281	6.22	281	7.57
2	796	17.62	1077	29.00
3	1258	27.84	2335	62.87
4	1087	24.05	3422	92.14
5	292	6.46	3714	100.00
Missing	805	17.81	4519	

**Table 2:** Descriptive statistics prior to standardization for statistically significant performance measures associated with hospitals’ overall quality star ratings.

Performance Measure (Identifier)	*n*	Mean	Median	Mode	SD	Min	Max
Hospital wide all-cause unplanned readmission (%) (READM_30_HOSP_WIDE)	4352	15.29	15.20	15.20	0.78	10.40	20.20
HCAHPS nurse communication (%) (H_COMP_1)	4109	80.08	80.00	79.00	5.58	20.00	99.00
HCAHPS responsiveness of hospital staff (%) (H_COMP_3)	4109	69.56	69.00	66.00	9.34	20.00	100.00
HCAHPS communication about medicines (%) (H_COMP_5)	4109	65.62	65.00	64.00	6.99	12.00	95.00
HCAHPS discharge information (%) (H_COMP_6)	4109	87.19	88.00	87.00	4.02	55.00	100.00
HCAHPS 3 item care transition measure (%) (H_COMP_7)	4109	53.17	53.00	53.00	6.98	15.00	98.00
HCAHPS Overall rating of hospital (%) (H_HSP_RATING)	4109	72.92	73.00	72.00	8.64	22.00	99.00
Pneumonia (PN) 30-day mortality rate (MORT_30_PN)	4017	15.87	15.80	15.40	1.97	9.00	24.80
Median time from ED arrival to ED departure for admitted ED patients (minutes) (ED_1b)	3971	273.18	256.00	220.00	109.75	64.00	1418.00
Admit decision time to ED departure time for admitted patients (minutes) (ED_2b)	3942	100.98	85.00	60.00	69.29	0.00	848.00
Abdomen CT use of contrast material (%) (OP_10)	3735	7.78	5.90	0.00	7.55	0.00	81.40
ED-patient left without being seen (%) (OP_22)	3616	1.56	1.00	1.00	1.59	0.00	18.00
Chronic obstructive pulmonary disease (COPD) 30-day mortality rate (MORT_30_COPD)	3533	8.40	8.30	8.10	1.11	4.90	14.40
Heart failure (HF) 30-day mortality rate (%) (MORT_30_HF)	3519	11.83	11.70	11.80	1.69	5.00	18.50
Patient safety and adverse events composite (%) (PSI_90)	3212	0.99	0.97	1.00	0.17	0.52	4.21
Endoscopy/polyp surveillance: colonoscopy interval for patients with a history of adenomatous polyps—avoidance of inappropriate use (%) (OP_30)	2795	90.92	97.00	100	14.24	0.00	100.00
Aspirin at arrival to ED (%) (OP_4)	2586	94.62	96.00	100	6.60	40.00	100.00
Acute Ischemic Stroke (STK) 30-Day Mortality Rate (MORT_30_STK)	2568	14.29	14.20	14.80	1.52	8.90	21.40
Acute myocardial infarction (AMI) 30-day mortality rate (MORT_30_AMI)	2318	13.20	13.10	12.80	1.22	8.90	18.70
External beam radiotherapy for bone metastases (%) (OP_33)	830	85.85	92.00	100.00	18.08	3.00	100.00

**Table 3:** Relative comparison of CMS model with the ordinal logistic regression approach.

Performance Measure Group	Performance Measure Identifier	Performance Measure Description (Data Collection Period)	CMS Loading Coefficient	Odds Ratio (95% CI)
Mortality	MORT_30_CABG	Coronary artery bypass graft (CABG) 30-day mortality rate (1 July 2014–30 June 2017)	0.33	
	PSI_4_SURG_COMP	Death rate among surgical inpatients with serious treatable complications (October 2015–30 June 2017)	0.28	
	MORT_30_AMI	Acute myocardial infarction (AMI) 30-day mortality rate (1 July 2014–30 June 2017)	0.51	0.86 (0.79, 0.93)
	MORT_30_STK	Acute ischemic stroke (STK) 30-day mortality rate (1 July 2014–30 June 2017)	0.48	0.86 (0.79, 0.93)
	MORT_30_PN	Pneumonia (PN) 30-day mortality rate (1 July 2014–30 June 2017)	0.66	0.46 (0.42, 0.50)
	MORT_30_COPD	Chronic obstructive pulmonary disease (COPD) 30-day mortality rate (1 July 2014–30 June 2017)	0.68	0.54 (0.49, 0.59)
	MORT_30_HF	Heart failure (HF) 30-day mortality rate (1 July 2014–30 June 2017)	0.71	0.47 (0.43, 0.52)
Readmission	EDAC_30_AMI	Excess days in acute care after hospitalization for acute myocardial infarction (1 July 2014–30 June 2017)	0.34	
	READM_30_CABG	Coronary artery bypass graft (CABG) 30-day readmission rate (1 July 2014–30 June 2017)	0.32	
	READM_30_Hip_Knee	Hospital-level 30-day all-cause risk- standardized readmission rate (RSRR) following elective total hip arthroplasty (THA)/total knee arthroplasty (TKA) (1 July 2014–30 June 2017)	0.41	
	EDAC_30_PN	Excess days in acute care after hospitalization for pneumonia (PN) (1 July 2014–30 June 2017)	0.44	
	EDAC_30_HF	Excess days in acute care after hospitalization for heart failure (1 July 2014–30 June 2017)	0.45	
	READM_30_COPD	Chronic obstructive pulmonary disease (COPD) 30-day readmission rate(1 July 2014–30 June 2017)	0.55	
	READM_30_HOSP_WIDE	Hospital wide all-cause unplanned readmission (1 July 2014–30 June 2017)	1.00	<0.001 (<0.001, <0.001)
	READM_30_STK	Stroke (STK) 30-day readmission rate (1 July 2014–30 June 2017)	0.53	
	OP_32	Facility seven-day risk-standardized hospital visit rate after outpatient colonoscopy (1 January 2017–31 December 2017)	−0.01	
Safety of Care	HAI_1	Central-line associated bloodstream infection (CLABSI) (1 April 2017–31 March 2018)	0.01	
	HAI_2	Catheter-associated urinary tract infection (CAUTI) (1 April 2017–31 March 2018)	0.01	
	HAI_6	Clostridium difficile (C. difficile) (1 April 2017–31 March 2018)	0.03	
	HAI_5	MRSA bacteremia (1 April 2017–31 March 2018)	0.04	
	HAI_3	Surgical site infection from colon surgery (SSI-colon) (1 April 2017–31 March 2018)	0.05	
	HAI_4	Surgical site infection from abdominal hysterectomy (SSI-abdominal hysterectomy) (1 April 2017–31 March 2018)	0.07	
	COMP_HIP_KNEE	Hospital-level risk-standardized complication rate (RSCR) following elective primary total hip arthroplasty (THA) and total knee arthroplasty (TKA) (1 April 2014–31 March 2017)	0.20	
	PSI_90	Patient safety and adverse events composite (1 October 2015–30 June 2017)	0.90	0.14 (0.12, 0.15)
Patient Experience	H_CLEAN_HSP	HCAHPS cleanliness of hospital environment (1 April 2017–31 March 2018)	0.69	
	H_COMP_6	HCAHPS discharge information (1 April 2017–31 March 2018)	0.68	1.40 (1.26, 1.57)
	H_QUIET_HSP	HCAHPS quietness of hospital environment (1 April 2017–31 March 2018)	0.71	
	H_COMP_3	HCAHPS responsiveness of hospital staff (1 April 2017–31 March 2018)	0.75	0.82 (0.70, 0.95)
	H_COMP_2	HCAHPS doctor communication (1 April 2017–31 March 2018)	0.74	
	H_COMP_5	HCAHPS communication about medicines (1 April 2017–31 March 2018)	0.75	1.21 (1.06, 1.38)
	H_COMP_1	HCAHPS nurse communication (1 April 2017–31 March 2018)	0.83	1.93 (1.69, 2.29)
	H_RECMND	HCAHPS willingness to recommend hospital (1 April 2017–31 March 2018)	0.86	
	H_COMP_7	HCAHPS 3 item care transition measure (1 April 2017–31 March 2018)	0.87	1.55 (1.34, 1.79)
	H_HSP_RATING	HCAHPS overall rating of hospital (1 April 2017–31 March 2018)	0.93	2.61 (2.22, 3.05)
Efficient Use of Medical Imaging	OP_13	Cardiac imaging for preoperative risk assessment for non-cardiac low-risk surgery (1 July 2016–30 June 2017)	0.01	
	OP_14	Simultaneous use of brain computed tomography (CT) and sinus CT (1 July 2016–30 June 2017)	0.02	
	OP_8	MRI Lumbar Spine for Low Back Pain (1 July 2016–30 June 2017)	0.01	
	OP_11	Thorax CT Use of Contrast Material (1 July 2016–30 June 2017)	0.29	
	OP_10	Abdomen CT Use of Contrast Material (1 July 2016–30 June 2017)	0.68	0.71 (0.65, 0.77)
Timeliness of Care	OP_3b_2	Median Time to Transfer to Another Facility for Acute Coronary Intervention (1 April 2017–31 March 2018)	0.15	
	OP_5	Median time to ECG (1 April 2017–31 March 2018)	0.18	
	ED_2b	Admit decision time to ED departure time for admitted patients (1 April 2017–31 March 2018)	0.78	0.84 (0.71, 0.99)
	OP_18b	Median time from ED arrival to ED departure for discharged ED patients(1 April 2017–31 March 2018)	0.80	
	ED_1b	Median time from ED arrival to ED departure for admitted ED patients (1 April 2017–31 March 2018)	0.83	0.72 (0.59, 0.88)
	OP_20	Door to diagnostic evaluation by a qualified medical professional (1 April 2017–31 March 2018)	0.42	
	OP_21	ED median time to pain management for long bone Fracture (1 April 2017–31 March 2018)	0.38	
Effectiveness of Care	PC_01	Elective delivery prior to 39 completed weeks gestation: percentage of babies electively delivered prior to 39 completed weeks gestation (1 April 2017–31 March 2018)	0.14	
	VTE_6	Hospital acquired potentially preventable venous thromboembolism (1 April 2017–31 March 2018)	0.17	
	IMM_2	Influenza immunization for patients (1 October 2017–31 March 2018)	0.33	
	OP_33	External beam radiotherapy for bone metastases (1 January 2017–31 December 2017)	0.34	1.13 (1.04, 1.23)
	OP_23	ED head CT or MRI scan results for acute ischemic stroke or hemorrhagic stroke who received head CT or MRI scan interpretation within 45 minutes of arrival (1 April 2017–31 March 2018)	0.40	
	SEP_1	Severe sepsis and septic shock(1 April 2017–31 March 2018)	0.49	
	OP_29	Endoscopy/polyp surveillance: appropriate follow-up interval for normal colonoscopy in average risk patients (1 January 2017–31 December 2017)	0.47	
	OP_22	ED patient left without being seen (1 January 2017–31 December 2017)	0.46	0.85 (0.78, 0.93)
	OP_30	Endoscopy/polyp surveillance: colonoscopy interval for patients with a history of adenomatous polyps—avoidance of inappropriate use (1 January 2017–31 December 2017)	0.62	1.14 (1.05, 1.23)
	IMM_3	Healthcare personnel influenza vaccination (1 October 2017–31 March 2018)	0.02	
	OP_4	Aspirin at arrival to the emergency department (ED) (1 April 2017–31 March 2018)	0.39	1.14 (1.05, 1.23)

## Data Availability

Data is available from the sources listed in the bibliography.
